# Active and passive tobacco and nicotine product exposure and asthma control in adolescents: a retrospective study from a pediatric allergy clinic

**DOI:** 10.3389/fped.2026.1952212

**Published:** 2026-10-28

**Authors:** Ozge Kangalli Boyacioglu, Ozgen Soyoz, Semiha Bahceci

**Affiliations:** Department of Pediatric Allergy and Immunology, Çiğli Training and Research Hospital, İzmir Bakırçay University, İzmir, Türkiye

**Keywords:** adolescent, asthma, asthma control, electronic cigarette, passive smoking, spirometry, tobacco use, vaping

## Abstract

**Background:**

Active tobacco and nicotine product use may adversely affect asthma in adolescents, but evidence from pediatric allergy clinics simultaneously evaluating active use, passive smoke exposure, asthma control, respiratory symptoms, and lung function remains limited. We examined associations between tobacco/nicotine exposure and asthma-related outcomes in adolescents receiving specialist allergy care.

**Methods:**

In this retrospective observational study, 238 adolescents aged 14–18 years with physician-diagnosed asthma were screened at a tertiary pediatric allergy clinic in İzmir, Türkiye, between January 2023 and July 2026; 208 met the eligibility criteria and were analyzed. Participants were classified at the most recent follow-up assessment as active tobacco/nicotine users (cigarettes, electronic cigarettes/e-puff, waterpipe, or multiple products; *n* = 50), passive-only exposed (*n* = 77), or unexposed (*n* = 81). Baseline spirometry was obtained at the initial visit, whereas Asthma Control Test (ACT) score, respiratory symptoms, asthma attacks during the preceding year, and treatment adherence were assessed at the most recent follow-up. Multivariable logistic regression evaluated uncontrolled asthma (ACT <20) and ≥1 asthma attack. Sensitivity analyses excluded active users with concurrent passive exposure and modeled ACT as a continuous outcome using multivariable linear regression with heteroskedasticity-robust standard errors.

**Results:**

Active users had lower ACT scores than passive-only and unexposed adolescents (median, 19 vs. 22 and 21; *p* < 0.001), and uncontrolled asthma occurred in 62.0%, 29.9%, and 34.6%, respectively (*p* < 0.001). After adjustment, active use was associated with uncontrolled asthma (adjusted odds ratio [aOR], 3.15; 95% confidence interval [CI], 1.45–6.81; *p* = 0.004), whereas passive-only exposure was not (aOR, 0.82; 95% CI, 0.42–1.62; *p* = 0.574). Active use was also associated with ≥1 asthma attack in the primary model (aOR, 2.20; 95% CI, 1.02–4.76; *p* = 0.044), but this estimate was not robust after excluding active users with concurrent passive exposure. In the continuous-ACT sensitivity analysis, active use was associated with a 2.43-point lower ACT score (adjusted *β*, −2.43; 95% CI, −3.87 to −0.99; *p* = 0.001).

**Conclusions:**

Among adolescents with asthma receiving specialist allergy care, current active tobacco/nicotine product use was associated with poorer asthma control. The association with recent asthma attacks was less robust, and passive-only exposure was not independently associated with the studied outcomes. Because exposure was self-reported and the study was retrospective and single-center, the findings should be interpreted as associations rather than causal effects.

## 1 Introduction

Asthma is a common chronic respiratory disease in adolescence, a period in which symptom burden, medication adherence, exposure to inhaled irritants, and health-related behaviors may influence disease control. Current asthma strategies recommend assessment of modifiable risk factors, including smoking and exposure to tobacco smoke, as part of routine asthma management (1).

The harmful effects of secondhand smoke exposure on childhood asthma are well established. Meta-analyses have linked secondhand smoke exposure with greater asthma morbidity and health care use, and population-based studies have reported a dose-related association with poor control and exacerbations (2, 3). However, the magnitude of this association varies across settings, partly because exposure intensity, location, duration, co-exposures, and the method used to ascertain exposure differ substantially between studies.

Adolescents are also increasingly exposed to a broader range of nicotine products, including electronic cigarettes and dual-product use. In adolescent samples, e-cigarette use has been associated with asthma and asthma symptoms, while a recent prospective study reported increased wheeze, bronchitic symptoms, and shortness of breath among e-cigarette users after adjustment for cigarette smoking and other inhalational exposures (4–7). The clinical implications may be especially important for adolescents with established asthma, in whom combustible tobacco smoke and e-cigarette aerosol can add irritant, oxidant, and inflammatory exposures to an already vulnerable airway.

Most available evidence on adolescent tobacco or nicotine exposure and asthma has come from community-based or cross-sectional studies, including studies of electronic-cigarette use (4–7). Fewer clinic-based studies have simultaneously characterized active nicotine product use, passive smoke exposure, allergic comorbidity, spirometry, bronchodilator response, and validated asthma-control measures within the same cohort. We therefore compared respiratory symptoms and baseline lung-function measures across exposure groups and evaluated adjusted associations with two prespecified clinical outcomes: uncontrolled asthma and at least one asthma attack during the preceding year.

## 2 Materials and methods

### 2.1 Study design and participants

This retrospective observational study was conducted at the Pediatric Allergy and Immunology Clinic of Bakırçay University Çiğli Training and Research Hospital, İzmir, Türkiye. Medical records of adolescents aged 14–18 years with physician-diagnosed asthma who attended the clinic between January 2023 and July 2026 were screened. Eligibility required an initial outpatient evaluation and at least one subsequent follow-up visit during the study period.

For each included participant, the initial visit date, most recent follow-up date, and total number of outpatient visits during the observation period were additionally reviewed. Follow-up duration was calculated as the interval between the initial and most recent visits and is reported in months.

The diagnosis of asthma was established by pediatric allergy specialists according to the Global Initiative for Asthma (GINA) recommendations based on compatible clinical history and objective evidence of variable expiratory airflow limitation.

A total of 238 potentially eligible adolescents were screened. Thirty were excluded: 20 because they had only an initial visit and did not return for follow-up, and 10 because baseline spirometry was unavailable. The final analytic cohort therefore comprised 208 adolescents. Tobacco/nicotine exposure status and ACT data were available for all 208 included participants; no imputation was performed for the variables used in the primary regression analyses.

The study protocol was approved by the İzmir Bakırçay University Non-Interventional Clinical Research Ethics Committee (Decision No. 3125; Research No. 3113; July 16, 2026). Because of the retrospective design, informed consent was waived by the ethics committee.

### 2.2 Tobacco exposure assessment

Tobacco and nicotine exposure was routinely assessed at the initial outpatient visit and updated during follow-up as part of the clinical history. For the present analysis, participants were classified according to their documented exposure status at the most recent follow-up assessment, concurrent with ACT assessment. Exposure information was based on patient self-report documented in the medical record; a standardized tobacco questionnaire and biochemical verification such as cotinine measurement were not used.

Patients were categorized into three mutually exclusive groups:

- Active tobacco/nicotine users: adolescents reporting daily use of at least one tobacco or nicotine product at the most recent follow-up, including conventional cigarettes, electronic cigarettes/e-puff products, waterpipe, or multiple products;
- Passive-only exposure: adolescents reporting no daily active tobacco/nicotine product use but having at least one parent (mother and/or father) who smoked cigarettes at home;
- No exposure: adolescents reporting neither daily active tobacco/nicotine product use nor parental cigarette smoking at home.

When daily active tobacco/nicotine use and parental smoking at home coexisted, participants were classified in the active-use group for the primary analyses. Because exposure was obtained from routine retrospective clinical documentation rather than a standardized research questionnaire, exact question wording and response options were not consistently available. Frequency beyond the daily-use criterion, cumulative dose, duration, and exact initiation dates were not sufficiently complete for quantitative exposure analyses. All 50 adolescents classified as active tobacco/nicotine users reported daily use; no occasional or less-than-daily active users were identified in the passive-only or unexposed groups.

### 2.3 Clinical assessment

The following variables were extracted from electronic medical records: age, sex, allergic rhinitis, atopic dermatitis, family history of atopy, aeroallergen sensitization, total serum IgE concentration, peripheral blood eosinophil count, asthma symptoms, asthma attacks during the preceding year, treatment adherence, and spirometric measurements.

Total serum immunoglobulin E (IgE) was measured by enzyme-linked immunosorbent assay (ELISA) and expressed as kIU/L.

Asthma control was evaluated at the most recent outpatient visit using the validated Asthma Control Test (ACT), a five-item patient-reported measure of asthma control. The Turkish ACT has demonstrated reliability and validity, and an ACT score <20 was used to define uncontrolled asthma (8, 9). An asthma attack was defined as worsening asthma symptoms requiring presentation to a healthcare facility. The number of such attacks during the preceding 12 months was obtained from the clinical record at the most recent follow-up and was analyzed as none versus at least one attack.

Treatment adherence was assessed retrospectively from longitudinal clinical records based on documented regular use of prescribed controller medication and regular attendance at scheduled follow-up visits. Asthma-control status itself was not used to define adherence.

### 2.4 Pulmonary function testing

Spirometry was performed at the initial outpatient visit using an EasyOne Connect spirometry system (ndd Medical Technologies, Zurich, Switzerland) by trained personnel in accordance with American Thoracic Society/European Respiratory Society technical standards (10).

Percent-predicted values of FEV₁, FVC, FEV₁/FVC, and FEF25–75 were recorded.

Bronchodilator reversibility was considered positive when post-bronchodilator FEV₁ increased by ≥12% and ≥200 mL compared with baseline, consistent with the criterion used in the clinical records during the study period (11). FEV₁ denotes forced expiratory volume in 1 s, FVC forced vital capacity, FEV₁/FVC the expiratory volume-to-capacity ratio, and FEF25–75 the forced expiratory flow between 25% and 75% of FVC.

### 2.5 Statistical analysis

Statistical analyses were performed using IBM SPSS Statistics version 26.0 (IBM Corp., Armonk, NY, USA).

Continuous variables were assessed for normality using visual inspection of histograms and the Shapiro–Wilk test. As most continuous variables were not normally distributed, data are presented as median and interquartile range (IQR).

Comparisons among the three exposure groups were performed using the Kruskal–Wallis test for continuous variables. When the overall test was statistically significant, pairwise comparisons were performed using Dunn's *post-hoc* test with Holm correction for multiple comparisons.

Categorical variables were compared using Pearson's chi-square test when expected cell frequencies were adequate and the Fisher–Freeman–Halton exact test when sparse cells made the asymptotic chi-square approximation inappropriate. The test used for each categorical comparison is identified in the table footnotes where relevant.

Two multivariable logistic regression models were constructed to evaluate the independent association between tobacco exposure and two outcomes: uncontrolled asthma (ACT <20) and at least one asthma attack during the preceding year.

The **unexposed group** served as the reference category.

Covariates were prespecified on clinical grounds before model fitting and were not selected according to univariable *p* values. The same available covariates were retained in both primary models: age, sex, allergic rhinitis, atopic dermatitis, family history of atopy, and treatment adherence. These variables were treated as clinically relevant adjustment variables rather than as a claim that each represented a proven causal confounder. Residual confounding by unmeasured factors was considered explicitly in the Discussion.

Adjusted odds ratios (aORs) with 95% confidence intervals (95% CIs) were calculated.

All 208 included participants had complete data for exposure group, the two regression outcomes, and the prespecified covariates; therefore, all were included in the primary regression analyses and no imputation was performed.

A two-sided ***p* value <0.05** was considered statistically significant.

Sensitivity analyses were conducted to assess robustness of the exposure findings. First, the 21 active users who also had passive smoke exposure were excluded and the primary models were repeated. Second, ACT was analyzed as a continuous outcome using multivariable linear regression with the same adjustment variables and heteroskedasticity-robust (HC3) standard errors. Variance inflation factors were examined to assess multicollinearity.

## 3 Results

### 3.1 Participant characteristics

A total of 238 adolescents were screened for eligibility. Thirty were excluded (20 with only an initial visit and no follow-up and 10 without baseline spirometry), leaving 208 adolescents in the final analysis (Figure 1). Of these, 50 (24.0%) were classified as active tobacco/nicotine product users, 77 (37.0%) had passive-only exposure, and 81 (38.9%) had no reported exposure. Among active users, 48 (96.0%) reported cigarette smoking, 13 (26.0%) electronic cigarette/e-puff use, one (2.0%) waterpipe use, and 12 (24.0%) multiple-product use. Twenty-one active users also had passive smoke exposure and were retained in the active-use group in the primary analysis.

**Figure 1:**
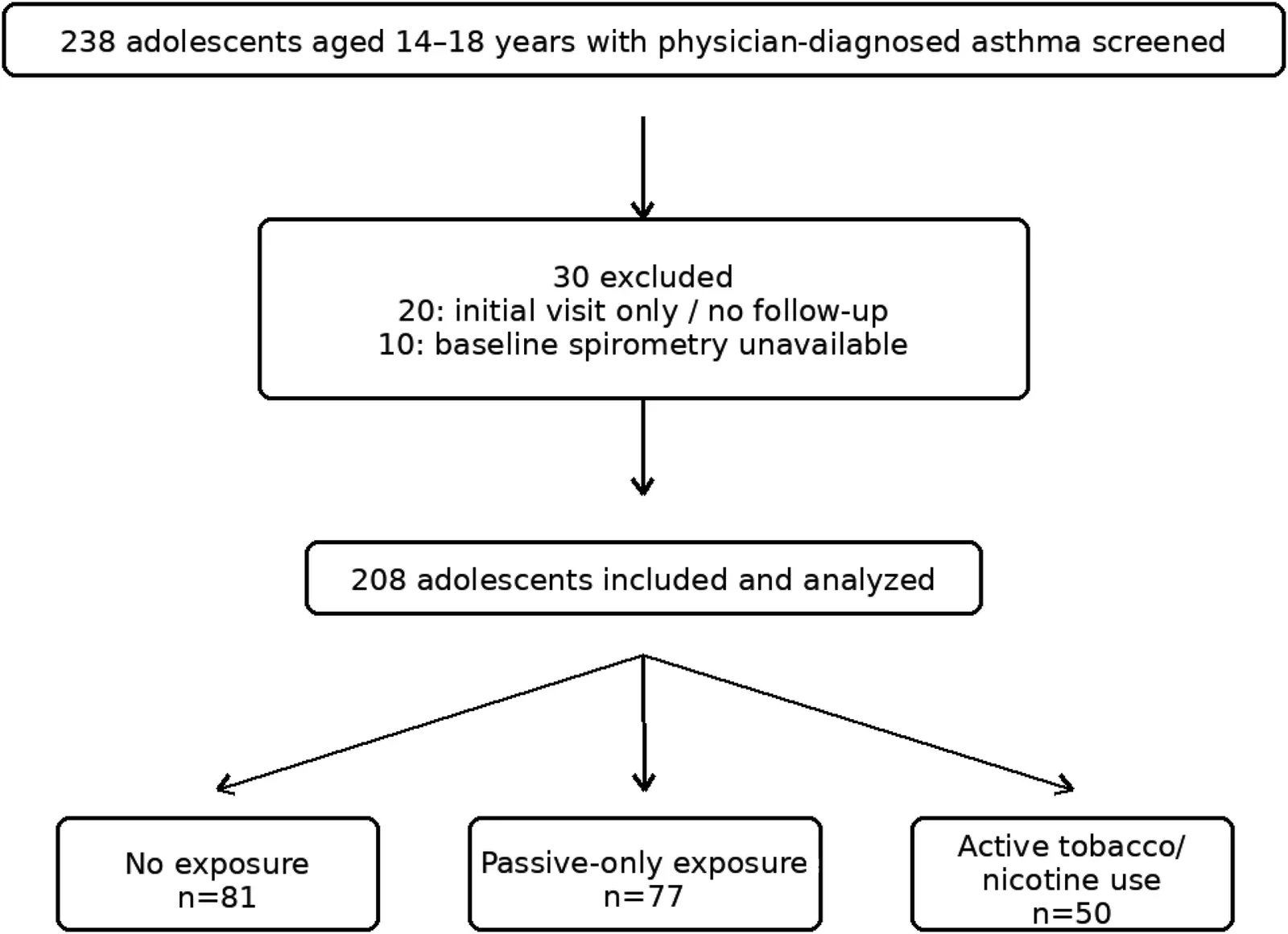
Participant screening and inclusion flow.

Across the 208 included adolescents, the median follow-up duration was 19.2 months (IQR, 12.5–31.3; range, 1.0–44.5 months), and the median number of outpatient visits was 8 (IQR, 5–12; range, 3–20). Follow-up dates and visit counts were available for all included participants.

Age differed across the exposure groups (overall *p* = 0.001). Active users were older than both adolescents with passive-only exposure (Holm-adjusted *p* = 0.001) and unexposed adolescents (Holm-adjusted *p* = 0.008), whereas the passive-only and unexposed groups did not differ. Sex distribution, allergic rhinitis, atopic dermatitis, family history of atopy, and treatment adherence were comparable across groups (Table 1).

**Table 1:** Demographic and selected clinical characteristics by exposure group.

Characteristic	No exposure (*n* = 81)	Passive only (*n* = 77)	Active use (*n* = 50)	*p* value
Age, years, median (IQR)	16.0 (14.0–17.0)	15.0 (14.0–17.0)	16.5 (15.0–17.0)	0.001
Female sex, *n* (%)	36 (44.4)	37 (48.1)	23 (46.0)	0.902
Allergic rhinitis, *n* (%)	67 (82.7)	60 (77.9)	43 (86.0)	0.494
Atopic dermatitis, *n* (%)	4 (4.9)	4 (5.2)	0 (0.0)	0.311
Family history of atopy, *n* (%)	45 (55.6)	35 (45.5)	21 (42.0)	0.253
Good treatment adherence, *n* (%)	54 (66.7)	54 (70.1)	27 (54.0)	0.162

Continuous variables were compared using the Kruskal–Wallis test. Pearson's chi-square test was used for sex, allergic rhinitis, family history of atopy, and treatment adherence; the Fisher–Freeman–Halton exact test was used for atopic dermatitis because of sparse cells. Exposure group and demographic/comorbidity variables reflect the most recent documented clinical assessment unless otherwise specified. In the overall cohort, median follow-up duration was 19.2 months (IQR, 12.5–31.3; range, 1.0–44.5) and the median number of visits was 8 (IQR, 5–12; range, 3–20).

Holm-adjusted pairwise comparisons for age: active use vs. passive only, *p* = 0.001; active use vs. no exposure, *p* = 0.008; passive only vs. no exposure, *p* = 0.458.

### 3.2 Asthma control, respiratory symptoms, and lung function

ACT scores differed across the exposure groups (overall *p* < 0.001), with lower scores among active users. Uncontrolled asthma was present in 31/50 active users (62.0%), compared with 23/77 adolescents with passive-only exposure (29.9%) and 28/81 unexposed adolescents (34.6%); the overall comparison was *p* < 0.001.

Night cough and sputum production were also more frequent among active users (both overall *p* < 0.001). At least one asthma attack during the preceding year was recorded in 32/50 active users (64.0%), 36/77 adolescents with passive-only exposure (46.8%), and 36/81 unexposed adolescents (44.4%); the overall unadjusted comparison was *p* = 0.073.

Baseline FEV1/FVC (% predicted) differed across groups (overall *p* = 0.001) and was lower in active users than in both the passive-only (Holm-adjusted *p* = 0.002) and unexposed groups (Holm-adjusted *p* = 0.003). FVC (% predicted) also differed in the overall analysis (*p* = 0.038), but none of the pairwise comparisons remained significant after Holm correction (both comparisons involving active users, adjusted *p* = 0.057). No statistically significant group differences were identified for FEV1, FEF25–75, or bronchodilator reversibility (Table 2).

**Table 2:** Asthma-related outcomes and baseline lung function by exposure group.

Outcome	No exposure (*n* = 81)	Passive only (*n* = 77)	Active use (*n* = 50)	*p* value
ACT score, median (IQR)	21 (18–24)	22 (19–24)	19 (15–20)	<0.001
Uncontrolled asthma (ACT <20), n/N (%)	28/81 (34.6)	23/77 (29.9)	31/50 (62.0)	<0.001
Night cough, *n* (%)	31 (38.3)	36 (46.8)	37 (74.0)	<0.001
Sputum production, *n* (%)	4 (4.9)	8 (10.4)	33 (66.0)	<0.001
≥1 asthma attack in preceding year, n/N (%)	36/81 (44.4)	36/77 (46.8)	32/50 (64.0)	0.073
FEV1, % predicted, median (IQR)	88 (80–98)	88 (77–102)	85 (74.2–96.8)	0.378
FEV1/FVC, % predicted, median (IQR)	101 (94–108)	102 (95–109)	94 (87–102)	0.001
FVC, % predicted, median (IQR)	93 (86–105)	95 (85–105)	90 (79–97.5)	0.038
FEF25–75, % predicted, median (IQR)	87 (70–106)	92 (73–105)	84.5 (67.2–101)	0.268
Positive bronchodilator reversibility, *n* (%)	31 (38.3)	40 (51.9)	18 (36.0)	0.119

Categorical outcomes were compared using Pearson's chi-square test. Holm-adjusted pairwise comparisons: ACT, active use vs. passive only, *p* < 0.001 and active use vs. no exposure, *p* = 0.001; FEV1/FVC, active use vs. passive only, *p* = 0.002 and active use vs. no exposure, *p* = 0.003. No FVC pairwise comparison remained significant after correction (both comparisons involving active use, *p* = 0.057).

ACT, Asthma Control Test; FEV1, forced expiratory volume in 1 s; FVC, forced vital capacity; FEF25–75, forced expiratory flow between 25% and 75% of FVC; IQR, interquartile range. ACT, symptoms, attack history, and treatment adherence were obtained at the most recent follow-up; spirometric measures and bronchodilator reversibility were obtained at the initial visit.

### 3.3 Adjusted associations with asthma outcomes

In the prespecified multivariable logistic regression model, with the unexposed group as the reference category, active tobacco/nicotine product use was independently associated with uncontrolled asthma (adjusted odds ratio [aOR], 3.15; 95% confidence interval [CI], 1.45–6.81; *p* = 0.004). Passive-only exposure was not independently associated with uncontrolled asthma (aOR, 0.82; 95% CI, 0.42–1.62; *p* = 0.574).

For at least one asthma attack during the preceding year, active tobacco/nicotine product use was also associated with higher odds in the primary adjusted model (aOR, 2.20; 95% CI, 1.02–4.76; *p* = 0.044), whereas passive-only exposure was not independently associated with this outcome (aOR, 1.11; 95% CI, 0.58–2.11; *p* = 0.752).

### 3.4 Sensitivity analyses

There were 82 uncontrolled-asthma events and 104 participants with at least one asthma attack. Multicollinearity was low (maximum variance inflation factor, 1.34). The full prespecified models, including estimates for all adjustment variables, are presented in Table 3.

**Table 3:** Fully adjusted multivariable logistic regression models for asthma outcomes.

Outcome/covariate	aOR	95% CI	*p* value
Uncontrolled asthma			
Exposure: passive only vs. no exposure	0.82	0.42–1.62	0.574
Exposure: active use vs. no exposure	3.15	1.45–6.81	0.004
Age, per year	0.97	0.77–1.22	0.780
Female sex	1.24	0.69–2.22	0.479
Allergic rhinitis	0.92	0.41–2.07	0.846
Atopic dermatitis	1.30	0.29–5.84	0.729
Family history of atopy	1.24	0.66–2.32	0.499
Good treatment adherence	0.58	0.31–1.06	0.075
≥1 asthma attack			
Exposure: passive only vs. no exposure	1.11	0.58–2.11	0.752
Exposure: active use vs. no exposure	2.20	1.02–4.76	0.044
Age, per year	0.94	0.75–1.18	0.607
Female sex	1.43	0.81–2.51	0.219
Allergic rhinitis	0.88	0.40–1.90	0.736
Atopic dermatitis	1.24	0.29–5.36	0.771
Family history of atopy	1.08	0.59–1.96	0.804
Good treatment adherence	0.45	0.25–0.82	0.009

aOR, adjusted odds ratio; CI, confidence interval. The unexposed group was the reference category for exposure. All listed covariates were entered simultaneously in each model.

After exclusion of the 21 active users who also had passive smoke exposure, active use remained associated with uncontrolled asthma (aOR, 4.08; 95% CI, 1.57–10.58; *p* = 0.004), whereas the estimate for at least one asthma attack was attenuated and imprecise (aOR, 1.69; 95% CI, 0.68–4.20; *p* = 0.260). When ACT was modeled continuously with robust standard errors, active use was associated with a 2.43-point lower ACT score compared with no exposure (adjusted *β*, −2.43; 95% CI, −3.87 to −0.99; *p* = 0.001), whereas passive-only exposure was not associated with ACT score (adjusted *β*, 0.53; 95% CI, −0.61 to 1.66; *p* = 0.363) (Table 4).

**Table 4:** Sensitivity analyses.

Sensitivity analysis	Outcome/contrast	Estimate	95% CI	*p* value
Exclude 21 active users with concurrent passive exposure	Uncontrolled asthma: active vs. no exposure	aOR 4.08	1.57–10.58	0.004
	≥1 asthma attack: active vs. no exposure	aOR 1.69	0.68–4.20	0.260
Continuous ACT model (HC3 robust SE)	Active use vs. no exposure	*β* −2.43	−3.87 to −0.99	0.001
	Passive only vs. no exposure	*β* 0.53	−0.61 to 1.66	0.363

aOR, adjusted odds ratio; ACT, Asthma Control Test; CI, confidence interval; HC3, heteroskedasticity-consistent robust standard errors. Sensitivity models used the same adjustment variables as the primary models.

## 4 Discussion

Current active tobacco/nicotine product use was associated with uncontrolled asthma after adjustment for age, sex, allergic comorbidities, family history of atopy, and treatment adherence. The association was also supported when ACT was analyzed as a continuous score and after exclusion of active users with concurrent passive exposure. In contrast, the evidence for recent asthma attacks was less robust: although the primary adjusted estimate was above the null, the confidence interval was close to 1.0 and the association was attenuated after exclusion of overlapping passive exposure. These findings therefore provide stronger evidence for an association with asthma control than for recent attacks and should not be interpreted as demonstrating causality.

Lower ACT scores among active users are clinically relevant because ACT is a validated measure of symptom control (8, 9). Current GINA guidance identifies smoking and electronic nicotine delivery systems among modifiable exposures considered in asthma management (1). Our findings support careful assessment of tobacco and nicotine exposure in adolescents with asthma, but this single retrospective study does not by itself establish that a specific screening or cessation strategy improves asthma outcomes.

The direction of the observed association cannot be determined from this retrospective study. Tobacco/nicotine use may worsen asthma control, but adolescents with poorer control may also differ in psychosocial, behavioral, environmental, or health-care factors that influence nicotine use. Recent reviews describe associations between adolescent vaping and asthma-related outcomes but also emphasize the predominance of cross-sectional evidence and the difficulty of causal inference (4, 12). The active category in this cohort included combustible cigarettes, electronic cigarettes/e-puff products, waterpipe, and multiple-product use. Product-specific estimates were not attempted because nearly all active users smoked cigarettes and the numbers of exclusive electronic-cigarette and waterpipe users were too small for reliable adjusted analyses.

Timing of measurement is important. Spirometry was performed at the initial visit, whereas exposure status used for the main analysis and ACT were determined at the most recent follow-up. Accordingly, baseline FEV1/FVC should not be interpreted as a consequence of the exposure status observed later in follow-up. The lower baseline ratio among adolescents ultimately classified as active users may reflect earlier exposure, pre-existing asthma-related airway characteristics, or other unmeasured differences. Prior longitudinal work in young adults with asthma has linked cigarette smoking with adverse lung-function outcomes (13). Repeated spirometry and prospectively time-updated exposure assessment would be required to evaluate lung-function trajectories.

Several biological processes could contribute to this clinical pattern. Combustible smoke delivers oxidants and reactive compounds that injure airway epithelium, weaken barrier function, promote mucus production, and impair mucociliary clearance. Electronic-cigarette aerosols differ chemically but may contain ultrafine particles, carbonyl compounds, metals, flavoring agents, and other oxidants. Their effects vary with the device, liquid composition, operating temperature, and pattern of use (14). In an asthmatic airway, epithelial injury and impaired clearance could amplify cough and sputum production without producing a measurable difference in bronchodilator reversibility at a single visit.

Smoke exposure may also alter the inflammatory setting in which asthma treatment acts. Cigarette smoke promotes neutrophil recruitment and innate immune activation; environmental irritants have been implicated in neutrophilic asthma, potentially through Th17/IL-17-related pathways (15). Recurrent epithelial injury, oxidative stress, and abnormal repair may favor airway hyperresponsiveness and remodeling. These mechanisms offer a coherent explanation for poorer control but were not measured here. The present data cannot define inflammatory phenotype, cytokine activity, or structural airway change.

Reduced corticosteroid responsiveness is another possible contributor. Oxidative and nitrative stress can diminish histone deacetylase 2 expression or activity, weakening corticosteroid-mediated suppression of inflammatory gene transcription (16). Adult clinical studies have reported attenuated responses to inhaled and oral corticosteroids among smokers with asthma (17, 18). Whether comparable impairment develops after shorter exposure during adolescence is uncertain, and adult data cannot be transferred directly to this age group. The possibility remains clinically relevant, particularly for regular or multiple-product users. Although treatment adherence was included in the adjusted models, adherence based on attendance and reported medication use cannot distinguish incomplete use from reduced pharmacological responsiveness.

The attack outcome warrants greater caution than the asthma-control outcome. Active use was associated with higher odds of at least one healthcare-attended asthma attack in the primary adjusted model, but the confidence interval was close to the null and the estimate was attenuated after exclusion of the 21 active users who also had passive exposure. This may reflect reduced precision after exclusion or contribution from overlapping exposure. Acute respiratory infections, allergen exposure, and access to acute care may also have influenced the recorded outcome. The findings therefore provide less robust evidence for the attack outcome than for asthma control. The attenuation may also be attributable, at least in part, to reduced statistical power resulting from the smaller analytic sample after exclusion of these 21 participants.

Passive-only exposure was not independently related to uncontrolled asthma or attacks. This differs from a substantial literature on secondhand smoke and childhood asthma. A systematic review and meta-analysis found greater health-care utilization among exposed children with asthma and an almost twofold likelihood of hospitalization for exacerbation (2). Adverse outcomes have also been reported among African-American and Latino children, with variation according to exposure burden and population susceptibility (3). The null estimates in this cohort should not be interpreted as evidence that passive exposure is harmless.

From a clinical perspective, the findings reinforce the value of documenting tobacco and nicotine exposure during adolescent asthma follow-up, including cigarettes, electronic cigarettes/e-puff products, waterpipe, and passive smoke exposure. Because exposure in this study was self-reported and not biomarker-verified, confidential and repeated history taking may reduce, but cannot eliminate, exposure misclassification (19).

Any cessation counselling should follow established adolescent tobacco-control guidance (20) rather than be inferred from the effect estimates of this study alone. Prospective studies with quantitative exposure measures, biochemical verification, repeated ACT assessment, and longitudinal spirometry are needed to determine whether changes in tobacco/nicotine exposure are followed by changes in asthma control.

### 4.1 Strengths and limitations

The three-group design distinguished active use from passive-only exposure and no reported exposure within the same clinical setting. The evaluation combined validated ACT scores, respiratory symptoms, attack history, allergic comorbidities, treatment adherence, baseline spirometry, and bronchodilator reversibility. The analysis retained the full prespecified adjustment set and used sensitivity analyses that directly addressed exposure overlap and ACT dichotomization.

The retrospective design limits causal interpretation. Exposure and outcomes were obtained from existing records rather than measured according to a prospective protocol, leaving the analysis vulnerable to incomplete documentation and residual confounding. Tobacco or nicotine use was self-reported and was not verified using cotinine or another biomarker; underreporting and exposure misclassification are therefore possible. Inclusion required at least one follow-up visit because asthma control, attacks, treatment adherence, and current tobacco/nicotine exposure were assessed at follow-up; accordingly, adolescents with only an initial visit were not eligible for these analyses. This eligibility criterion should be considered when interpreting the findings. Although no occasional or less-than-daily users were identified in the passive-only or unexposed groups, exposure classification relied on self-report; therefore, underreporting of occasional tobacco/nicotine use cannot be excluded.

The timing of measurements creates a further constraint. Baseline spirometry was obtained at the initial visit, whereas the exposure classification used in the primary analysis, ACT, symptoms, adherence, and attack history were obtained from the most recent follow-up. Exposure was routinely reassessed during follow-up, allowing adolescents who initiated active use to be classified according to their current status at the ACT assessment; however, exact initiation dates, cumulative exposure, and intermediate exposure trajectories were not systematically available. The absence of longitudinal spirometry prevents assessment of individual lung-function trajectories. Treatment adherence was based on follow-up attendance and documented medication use rather than pharmacy dispensing, electronic monitoring, or a validated adherence scale.

Other determinants of nicotine use and asthma control may not have been captured. Socioeconomic circumstances, body mass index, ambient and household air pollution, psychosocial stress, peer influences, other inhaled substances, and viral exposure could have contributed to the observed associations. Participants were recruited from a single tertiary pediatric allergy clinic in İzmir, Türkiye; their comorbidity profiles, referral patterns, prior counselling, and disease severity may differ from those of adolescents treated in other tertiary centers in İzmir or elsewhere in Türkiye, in primary care, or in community samples. Generalisability is therefore limited.

## 5 Conclusions

Current active tobacco/nicotine product use was associated with poorer asthma control among adolescents receiving specialist allergy care, including when ACT was analyzed as a continuous outcome. The estimate for recent asthma attacks was less robust, and passive-only exposure was not independently associated with the studied outcomes. These findings are observational and should not be interpreted as causal. Prospective multicenter studies with biomarker-verified, quantitative, and time-updated exposure assessment are needed to clarify temporal and product-specific relationships with asthma control and lung-function trajectories.

## Data Availability

The de-identified data supporting the findings of this study may be made available by the corresponding author upon reasonable request, subject to applicable ethical, institutional, and data protection requirements.
